# An unusual suspect: an uncommon human-specific synonymous coding variant within the *UGT1A6* gene explains a GWAS signal and protects against bladder cancer

**DOI:** 10.1186/gb-2011-12-s1-p19

**Published:** 2011-09-19

**Authors:** Wei Tang, Yi-Ping Fu, Jonine D Figueroa, Núria Malats, Montserrat Garcia-Closas, Nilanjan Chatterjee, Manolis Kogevinas, Dalsu Baris, Michael Thun, Jennifer L Hall, Immaculata De Vivo, Demetrius Albanes, Patricia Porter-Gill, Mark P Purdue, Laurie Burdett, Luyang Liu, Amy Hutchinson, Timothy Myers, Adonina Tardón, Consol Serra, Alfredo Carrato, Reina Garcia-Closas, Josep Lloreta, Alison Johnson, Molly Schwenn, Margaret R Karagas, Alan Schned, Amanda Black, Eric J Jacobs, W Ryan Diver, Susan M Gapstur, Jarmo Virtamo, David J Hunter, Joseph F Fraumeni, Stephen J Chanock, Debra T Silverman, Nathaniel Rothman, Ludmila Prokunina-Olsson

**Affiliations:** 1 Laboratory of Translational Genomics, Division of Cancer Epidemiology and Genetics, National Cancer Institute, Bethesda, MD 20892, USA; 2 Division of Cancer Epidemiology and Genetics, National Cancer Institute, Bethesda, MD 20892, USA; 3 Spanish National Cancer Research Centre, Madrid 28029, Spain; 4 Division of Genetics and Epidemiology, Institute of Cancer Research, London SW7 3RP, UK; 5 Centre for Research in Environmental Epidemiology (CREAL), Barcelona 08003, Spain; 6 Municipal Institute of Medical Research, Barcelona 08003, Spain; 7 CIBER Epidemiología y Salud (CIBERESP), Barcelona 08003, Spain; 8 National School of Public Health, Athens 11521, Greece; 9 Epidemiology Research Program, American Cancer Society, Atlanta, GA 30303, USA; 10 Lillehei Heart Institute, Department of Medicine, University of Minnesota, Minneapolis, MN 55455, USA; 11 Channing Laboratory, Department of Medicine, Brigham and Women’s Hospital, Boston, MA 02115, USA; 12 Core Genotype Facility, SAIC-Frederick, National Cancer Institute, Frederick, MD 21702, USA; 13 Universidad de Oviedo, Oviedo 33003, Spain; 14 Universitat Pompeu Fabra, Barcelona 08002, Spain; 15 Ramón y Cajal University Hospital, Madrid 28034, Spain; 16 Unidad de Investigación, Hospital Universitario de Canarias, La Laguna 38320, Spain; 17 Hospital del Mar-Institut Municipal d’Investigació Mèdica (IMIM), Universitat Pompeu Fabra, Barcelona 08003, Spain; 18 Vermont Cancer Registry, Burlington, VT 05401, USA; 19 Maine Cancer Registry, Augusta, ME 04333, USA; 20 Dartmouth Medical School, Hanover, NH 03755, USA; 21 Department of Urology, Washington University School of Medicine, St. Louis, MO 63110, USA; 22 National Institute for Health and Welfare, Helsinki 00271, Finland; 23 Department of Epidemiology, Program in Molecular and Genetic Epidemiology, Harvard School of Public Health, Boston, MA 02115, USA

## Background

A recent genome-wide association study (GWAS) of bladder cancer identified a single nucleotide polymorphism (SNP), rs11892031, within the *UGT1A* gene cluster on chromosome 2q37.1, as a novel risk factor. The *UGT1A* locus encodes nine UGT proteins, which belong to the phase II cellular detoxification system. UGTs are functionally important for the detoxification of aromatic amines, which are found in industrial chemicals and tobacco smoke and are known risk factors for bladder cancer. The UGT-encoding genes have exons 2 to 5 in common but have different first exons, which define the enzymatic activity and substrate specificity of the gene products.

## Methods and results

We sequenced all nine highly similar alternative first exons for the UGT-encoding genes of up to 2,000 individuals. We identified 26 known nonsynonymous and 17 known synonymous coding variants but no novel variants. Imputation based on the GWAS dataset, a combined reference panel of HapMap 3 and the 1000 Genomes Project, and a subset of GWAS samples genotyped for all of the identified coding variants generated data for 1,170 SNPs within the whole *UGT1A* region. Of these markers, the strongest association was detected for an uncommon protective genetic variant that explained the original GWAS signal (odds ratio (OR) = 0.55, 95% confidence interval (CI) = 0.44 to 0.69, *P* = 3.3 × 10^–7^ in 4,035 cases and 5, 284 controls; D′ = 0.96, *r*^2^ = 0.23 with rs11892031). No residual association in this region was detected after adjustment for this SNP. A typical genetic variant identified by GWAS for a common disease is expected to be a common allele (>10% minor allele frequency) that increases the disease risk. We show that the novel associated variant is an uncommon protective allele (1.14% in cases and 2.5% in controls). Interestingly, the risk allele (G) is conserved in 33 species, whereas the protective allele (T) is a human-specific variant. Even though this SNP is a synonymous coding variant, we show its association with quantitative mRNA expression of a specific functional splicing form of *UGT1A6*, probably through an exonic splicing enhancer.

## Conclusions

This study exemplifies that uncommon protective genetic variants are unusual suspects that may play important but underestimated functional roles in complex traits.

